# Application Effect of Bladder Function Training Combined with Kangaiping Pills on Permanent Bladder Stoma after Radical Prostatectomy

**DOI:** 10.1155/2022/6211543

**Published:** 2022-03-24

**Authors:** Kefu Sha, Yue Zhao, Deng Yang, Zhaoxia Song, Mingjun Zhao, Jieqing Gao, Tiejun Liu

**Affiliations:** Urology Surgery, Beijing Rehabilitation Hospital, Beijing 100144, China

## Abstract

**Objective:**

To investigate the application effect of bladder function training combined with Kangaiping pills on permanent bladder stoma after radical prostatectomy (RP).

**Methods:**

The clinical data of 80 patients with a permanent bladder stoma after RP in our hospital from December 2018 to December 2019 were retrospectively analyzed, and they were equally split into the experimental group (EG) and control group (CG) according to the odd and even hospitalization numbers. EG received bladder function training combined with Kangaiping pills while CG received routine nursing for permanent bladder stomas to compare the urodynamic indexes and quality of life (QOL) scores after intervention between the two groups.

**Results:**

Compared with CG, EG after intervention achieved an obviously higher number of patients with bladder function grade I (*∗*), higher urodynamic indexes (*P* < 0.001), a higher SF-36 score (*P* < 0.001), a lower LUTS score (*P* < 0.001), and a lower total incidence of postoperative adverse reactions (*P* < 0.05).

**Conclusion:**

Bladder function training combined with Kangaiping pills is a reliable method to improve the bladder function of patients with a permanent bladder stoma after RP. This intervention method greatly enhances the QOL of patients and reduces the risk of postoperative adverse reactions, which is recommended for clinical application.

## 1. Introduction

Prostate cancer (PC) is a common malignant tumor disease in men, with morbidity and mortality ranking second and sixth in all male malignant tumors worldwide [1, 2]. In recent years, with the acceleration of population aging in China, the harm of PC to Chinese men has been increasing [3]. With the continuous improvement of clinical surgical techniques in China, radical prostatectomy (RP) has become a common surgical approach in clinical practice, as well as a gold standard of surgery for PC [4, 5]. Since RP has changed the normal urination, a permanent bladder stoma is necessary to protect renal function and maintain normal water-electrolyte metabolism and homeostasis.

Cystostomy is mainly used in patients with prostatic hyperplasia who are unable to undergo surgery, urethrostenosis, urethrectomy due to urethrophyma, neurogenic bladder, and other diseases that require urine drainage [6]. Since patients with a permanent bladder stoma need to carry a fistula device for a long time and are prone to postoperative complications such as urinary tract infection and cystospasm, the implementation of necessary postoperative intervention measures is particularly critical to improving the prognosis of patients [7]. A study [8] has confirmed that effective bladder function training can make detrusor constantly move, which increases the flushing effect of urine, shortens the stay of bacteria, decreases the risk of infection, and plays a positive role in reducing postoperative adverse reactions. In recent years, the intensive research of traditional Chinese medicine (TCM) in cancer has found that the pathogenesis of PC is closely related to the kidney [9]. The combination of internal and external wind, cold, and toxins causes phlegm turbidity and cold coagulation to block blood vessels. Phlegm turbidity, blood stasis, cold coagulation, and cancer toxins mix with each other and cannot be eliminated, eventually leading to diseases after long-time accumulation. TCM is the treasure of Chinese medicine, which is of great value in the clinical treatment of a variety of diseases, with significant advantages in regulating human organ function and quality of life. Kangaiping pills, composed of more than ten herbs such as loosestrife and herba sarcandrae, can relieve stasis, remove pain, and clear away heat and toxic material [10], thus fighting against tumors and increasing body immunity, which has been confirmed in gastrointestinal tumors such as rectal cancer and gastric cancer [11]. At present, few reports have been found on the clinical efficacy of bladder function training combined with Kangaiping pills. In this study, eighty patients with a permanent bladder stoma after RP in our hospital were selected as the research subjects to explore the effect of this intervention method on the patients, reported as follows.

## 2. Materials and Methods

### 2.1. General Information

Eighty patients with a permanent bladder stoma after RP in our hospital were selected as the research subjects, and all patients completed relevant clinical examinations after admission. It was a retrospective analysis, the provision of patients' informed consent was waived upon the review of the hospital, and the trail met the Declaration of Helsinki (as revised in 2013) [12].

### 2.2. Inclusion and Exclusion Criteria

#### 2.2.1. Inclusion Criteria

The inclusion criteria were as follows: (1) patients who met the PC diagnostic criteria and were confirmed by pathological examinations, with an age ≤75 years; (2) patients who had clear consciousness, could move freely, and were willing to cooperate; and (3) patients who had no distant metastasis and no other serious complications.

#### 2.2.2. Exclusion Criteria

The exclusion criteria were as follows: (1) patients with other types of cancer; (2) patients who were discharged during the study; (3) patients suffering from serious chronic diseases; and (4) patients who were delirious and unable to express subjective wishes on their own.

### 2.3. Methods

Routine nursing for permanent bladder stomas was performed to the 40 patients in CG. The surgeons determined the patients' degree of recovery by observing their stoma status for the physicians to prepare medication and rehabilitation plans. The nurses explained the preoperative preparation, the importance, and specific steps of permanent cystostomy and introduced the characteristics and usage of different urostomy pouches and other auxiliary supplies to the patients and their families. The nurses instructed the patients to choose suitable stoma care supplies and carefully explained and demonstrated the replacement of urostomy pouches and precautions. They also carried out clinical health education, with diet and discharge guidance to the patients [13, 14].

Bladder function training combined with Kangaiping pills was performed to the 40 patients in EG. Bladder function training was implemented as follows. The patients were told to drink enough water to ensure the daily water intake of 1.5–2.0 L, and personalized programs for drinking water were formulated according to the daily eating habits of patients. In terms of trigger point urination training, the patients were instructed to separate their legs and imagine the sensation of urination while their inner thigh and suprapubic region were stimulated, and their feedback of the urination stimulation points was timely recorded. The stimulation was strengthened to promote detrusor contraction and activate autonomous urination reflex, and the training was performed once a day, five times a week, and consecutively for 4 weeks. According to th physician's prescription, the pharmacists gave patients the Kangaiping pills (manufacturer: Hebei Hongri Yaodu Pharmaceutical Co., Ltd.; NMPA approval no. Z46020009; specification: 1 g *∗* 18 bottles), with the dosage of 0.5–1.0 g each time and 3 times a day, and the dose could be controlled within 0.5 g at the first-time administration and then could be added gradually, and if patients felt distention in the stomach, the dose could be reduced appropriately. With 3 weeks as a course of treatment, the patients took 2 courses continuously.

### 2.4. Evaluation Indexes

#### 2.4.1. Classification of Bladder Function

The residual urine volume under B ultrasound after extubation was recorded in both groups, with the volume <50 ml as grade I, 50–100 ml as grade II, >100 ml as grade III, and severe dysuria as grade IV.

#### 2.4.2. Urodynamic Indexes

The urodynamic equipment (manufacturer: Shanghai Hanfei Medical Device Co., Ltd.; model: Nidoc 970A) was used to measure the Valsalva leak point pressure (LPP) and maximum urethral closure pressure (MUCP) of both groups after intervention. The international lower urinary tract symptom score (LUTS) [15] was used to evaluate the urination status of both groups after the intervention, including 7 items, with each item scoring 5 points and a total score of 35 points. A higher score represented worse urination function. The SF-36 scale [16] was used to evaluate the quality of life (QOL) of both groups after the intervention, including physical pain, physiological function, and emotional function, with a total score of 100 points. A higher score demonstrated better QOL. The incidence of postoperative adverse reactions in both groups was recorded.

### 2.5. Statistical Methods

The data were processed by the professional statistical software SPSS26.0 and graphed by GraphPad Prism 7 (GraphPad Software, San Diego, USA). The enumeration data were tested by *X*^2^ and expressed as (*n* (%)), while the measurement data were tested by *t*-test and expressed as Mean ± SD. When *P* < 0.05, the differences were statistically significant.

## 3. Results

### 3.1. Clinical Data

No notable differences were observed in the average age, family income, and residence of patients between the two groups (*P* < 0.001) (see Table 1).

### 3.2. Comparison of Changes in Bladder Function Classification

After intervention, the numbers of cases with bladder function grade I, II, III, and IV were, respectively, 27, 11, 2, and 0 in EG and were, respectively, 18, 13, 6, and 3 in CG. The numbers of cases with bladder function grade I were significantly different between the two groups (*P* < 0.05), as presented in Table 2.

### 3.3. Urodynamic Indexes

Table 3 presented obviously higher LPP and MUCP values in EG than in CG after intervention (*P* < 0.001).

### 3.4. LUTS Scores

**Figure 1:**
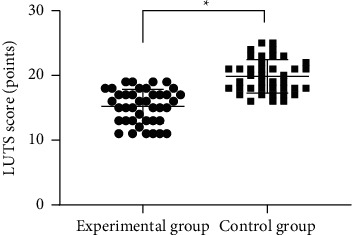
Comparison of LUTS scores between the two groups after intervention (mean ± SD). *Note.* The abscissa represented EG and CG, and the ordinate represented the LUTS score (points). The average LUTS scores of EG and CG after intervention were (15.23 ± 2.63) and (19.85 ± 2.59), respectively. ^*∗*^represents a notable difference in the average LUTS scores after intervention between the two groups (*t* = 7.916, *P* < 0.001).

In terms of urinary function, the average LUTS score in EG after intervention was remarkably lower compared with CG (*P* < 0.001) (see Figure 1).

### 3.5. QOL

**Figure 2:**
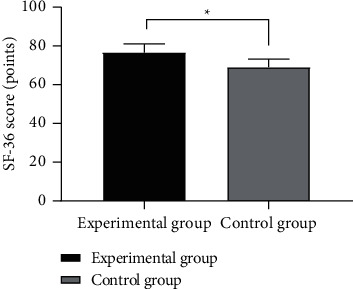
Comparison of SF-36 scores between the two groups after intervention (mean ± SD). *Note.* The abscissa represented EG and CG, and the ordinate represented the SF-36 score (points). The SF-36 scores of EG and CG after intervention were (76.60 ± 4.44) and (69.00 ± 4.21), respectively. ^*∗*^represents a notable difference in the SF-36 scores after intervention between the two groups (*t* = 7.856, *P* < 0.001).

In terms of QOL, after intervention, the total QOL score in EG was notably higher compared with CG (*P* < 0.001) (see Figure 2).

### 3.6. Incidence of Postoperative Adverse Reactions

Peristomal skin infection is a common type of postoperative complication in patients undergoing RP. After surgery, 1 case in EG had peristomal skin infection, and there were 2 cases with urinary tract obstruction, 1 case with cystospasm, and 3 cases with peristomal skin infection in CG. Compared with CG, EG achieved a lower total incidence of postoperative adverse reactions (*P* < 0.05), as shown in Table 4.

## 4. Discussion

In China, prostate cancer (PC) has become one of the malignant tumors with the fastest rising morbidity and mortality in recent years due to the gradual aging of population structure, westernization of living and eating habits, and the application and promotion of prostate-specific antigen (PSA) screening [17]. PC progresses rapidly and can easily pose a threat to the life safety of patients without timely treatment. With the advantages of the open surgical field and easy operation, radical prostatectomy (RP) is currently one of the most effective methods for PC treatment and can complete pelvic lymphadenectomy via the same approach, which is favored by doctors and patients. Permanent cystostomy is the surgical treatment for urethral obstruction with suprapubic cystostomy to achieve permanent urinary diversion, which is clinically applicable to many diseases such as total urinary tract resection of urethral tumors and neurogenic bladder [18]. However, many adverse effects such as blocked stoma tube and skin infection around the stoma can occur after cystostomy. Combined with the inconvenience to the patients' mobility caused by a permanent bladder stoma, these effects seriously affect QOL of the patients and arouse their resistance to treatment, thus hindering postoperative rehabilitation.

A study [19] has confirmed that early bladder function training after surgery can enhance the pressure of the sphincter, pelvic floor muscle, and urethra, promote the recovery of bladder function, improve urination, and help patients return to normal life as soon as possible. With the continuous development of modern Chinese medicine, traditional Chinese medicine (TCM) has been widely used in treating various types of malignant tumors. Kangaiping pills, composed of loosestrife, barbated skullcup herb, hedyotis, Indian mock strawberry, actinidia chinensis planch, toad venom, all-grass of Bluecalyx Japanese Rabdosia, sarcandra, common bluebeard herb, and herba selaginellae doederleinii, conform to the TCM concept of strengthening the body and restoring normal function in clinical treatment [20]. The pills can effectively activate the anticancer factors of the human body, which plays an important role in improving the autoimmunity of patients with malignant tumors, and are often used to treat the digestive system malignant tumors such as gastric cancer, esophageal cancer, and rectal cancer [21, 22].

In this study, the number of cases with bladder function grade I was markedly higher in EG than in CG after intervention (*P* < 0.05), suggesting that bladder function training combined with Kangaiping pills can effectively improve the bladder function of patients with a permanent bladder stoma after RP. The reason may be that bladder function training adopted in this study can stimulate the inner thigh and suprapubic region to find the urination stimulation points, promote detrusor contraction, and activate spontaneous micturition reflex by strengthening stimulation, while the application of Kangaiping pills can effectively inhibit the metastasis and proliferation of tumor cells, protect the hematopoietic function of patients, improve clinical symptoms, and indirectly accelerate the improvement of bladder function [23]. In terms of urodynamic indexes and voiding condition, patients who received bladder function training combined with Kangaiping pills intervention had more significant results, because bladder function training can effectively stimulate the recovery of bladder function, help regulate the periodic contraction rhythm of the bladder, train the awareness of urination, and maintain the maximum capacity and pressure of the bladder, and Kangaiping pills can better promote the reverse differentiation of tumor cells, control the continued spread of metastases, reduce symptoms, improve the functional condition of the body, and promote the improvement of urination function. In the clinical education of cystostomy, bladder function training should be actively carried out in addition to increasing water intake, keeping stoma clean and dry, and timely replacing bladder fistula. The stoma left in the bladder and open to the outside world provides the possibility for bacteria to enter the bladder [24]. In addition, as a foreign body, the fistula remaining in the bladder for a long time greatly damages the normal bladder environment. Therefore, the patients are prone to various adverse reactions. This study demonstrated that EG had a notably lower total incidence of adverse reactions after intervention compared with CG (*P* < 0.05), illustrating that the bladder function training combined with Kangaiping pills can effectively reduce the incidence of postoperative adverse reactions in patients. It is speculated that bladder function training can effectively stimulate detrusor activity, enhance the flushing effect of urine, and greatly shorten the stay of bacteria in the bladder, thereby reducing the possibility of bacterial infection, avoiding the spasm due to the stimulation of fistula to the bladder mucosa to a certain extent, and promoting urination [25]. In addition, in terms of QOL, the SF-36 score in EG after the scientific intervention was obviously higher than that in CG (*P* < 0.001), demonstrating that the implementation of bladder function training for patients with a permanent bladder stoma can help improve urination, reduce the occurrence of adverse reactions, and enhance QOL. This study also has some inadequacies, such as a small sample size and a lack of research about the effect of age and bladder function before treatment on the findings of this study due to the limitations of research conditions. Therefore, in future treatment, the bladder function of such patients should be evaluated, and more scientific and systematic postoperative intervention measures should be formulated to reduce the adverse reactions after colostomy, alleviate the pain, relieve the economic burden, and improve QOL of patients.

## Figures and Tables

**Table 1:** Comparison of clinical data (*n* = 40).

Items	EG	CG	*X* ^2^/*t*	*P*
Average age (mean ± SD, years)	65.83 ± 3.46	65.79 ± 3.52	0.051	0.959
BMI (mean ± SD, kg/m^2^)	21.72 ± 1.35	21.76 ± 1.42	0.129	0.898
Hypertension			0.202	0.653
Yes	17 (42.50%)	19 (47.50%)		
No	23 (57.50%)	21 (52.50%)		
Diabetes			0.219	0.640
Yes	11 (27.50%)	13 (32.50%)		
No	29 (72.50%)	27 (67.50%)		
Family income			0.205	0.651
≥3000 yuan/(month/person)	16 (40.00%)	18 (45.00%)		
<3000 yuan/(month/person)	24 (60.00%)	22 (55.00%)		
Residence (*n* (%))			0.453	0.501
Urban area	17 (42.50%)	20 (50.00%)		
Rural area	23 (57.50%)	20 (50.00%)		
Education (*n* (%))				
College degree or above	2 (5.00%)	3 (7.50%)	0.213	0.644
Senior high school	6 (15.00%)	4 (10.00%)	0.457	0.499
Junior high and below	32 (80.00%)	33 (82.00%)	0.082	0.775

**Table 2:** Comparison of changes in bladder function classification (*n* (%)).

Group	*n*	Grade I	Grade II	Grade III	Grade IV
EG	40	27 (67.50)	11 (27.50)	2 (5.00)	0 (0.00)
CG	40	18 (45.00)	13 (32.50)	6 (15.00)	3 (7.50)
*X* ^2^		4.114	0.219	2.222	3.117
*P*		<0.05	0.640	0.136	0.077

**Table 3:** Comparison of urodynamic indexes after intervention (mean ± SD, kPa).

Group	*n*	LPP	MUCP
EG	40	11.82 ± 1.35	37.64 ± 3.27
CG	40	9.26 ± 1.17	29.29 ± 3.31
*T*		9.063	11.350
*P*		<0.001	<0.001

**Table 4:** Comparison of occurrence of postoperative adverse reactions (*n* (%)).

Group	*n*	Urinary tract obstruction	Cystospasm	Skin infection around the stoma	Total incidence
EG	40	0 (0.00)	0 (0.00)	1 (2.50)	2.50% (1/40)
CG	40	2 (5.00)	1 (2.50)	3 (7.50)	15.00 (6/40)
*X* ^2^					3.914
*P*					<0.05

## Data Availability

The data to support the findings of this study are available on reasonable request from the corresponding author.

## Abbreviations:

EG: Experimental group
CG: Control group
RP: Radical prostatectomy
QOL: Quality of life
PC: Prostate cancer
TCM: Traditional Chinese medicine
MUCP: Maximum urethral closure pressure
LPP: Valsalva leak point pressure
LUTS: Lower urinary tract symptom score
PSA: Prostate-specific antigen.
